# Lymphoma as a Rare Cause of Ureteral Obstruction: A Case Report

**DOI:** 10.1155/criu/1563744

**Published:** 2026-08-21

**Authors:** Michael J. Markel, Michael Chang, Elisabeth S. Rindner, Jenna Dickman, Krishnan Venkatesan, Nathan M. Shaw

**Affiliations:** ^1^ Department of Urology, MedStar Georgetown University Hospital/Washington Hospital Center, Washington, DC, USA, medstarwashington.org; ^2^ Georgetown University School of Medicine, Washington, DC, USA, georgetown.edu; ^3^ Department of Urology, Washington DC Veterans Affairs Medical Center, Washington, DC, USA

**Keywords:** case report, lymphoma, obstructive uropathy, ureteral obstruction

## Abstract

Extranodal marginal zone lymphoma involving the urinary system is a rare manifestation of lymphoma. Ureteric obstruction by this subtype of lymphoma can mimic other more common genitourinary or retroperitoneal conditions. The current case describes a 42‐year‐old female who presented with abdominal pain and hydronephrosis without findings supportive of renal or ureteric calculus. Diagnostic work‐up was unremarkable, even with the employment of endoscopic evaluation. The patient′s ureter was dilated and she underwent both stenting and eventual percutaneous nephrostomy tube placement. Ultimately, she underwent surgical intervention with ureteral reimplantation and associated histopathological analysis was able to definitively diagnose a low‐grade B‐cell extranodal marginal zone lymphoma. The present case highlights the diagnostic challenges associated with this rare etiology of urinary obstruction. It also emphasizes the importance of considering atypical malignancy in the differential diagnosis of recurrent ureteral obstruction, especially when traditional radiological imaging and clinical assessment are unrevealing.

## 1. Introduction

Lymphomas are a heterogeneous group of hematologic malignancies that most commonly involve nodal structures but can present extranodally in disparate organ systems. Although genitourinary involvement by lymphoma is uncommon, ureteral involvement and consequent obstruction have been reported in case reports and small series, underscoring the need for awareness among clinicians and pathologists when evaluating obstructive uropathy of unclear etiology [1–6]. Unfortunately, diagnostic work‐up for patients with ureteral lymphoma continues to be a significant challenge due to various causes of obstruction that mimic its presentation [7, 8]. Ureteric calculi, strictures, and urothelial carcinoma are some etiologies that present in a similar fashion on modalities such as ureteroscopy or pyelogram [7, 8]. The ureter represents a particularly rare site for primary involvement, with primary ureteral lymphomas described infrequently and often mimicking more common urothelial or extrinsic compressive processes [1, 3, 5]. Here, we describe an unusual presentation of extranodal marginal zone lymphoma causing acute ureteral obstruction. In our patient case, she had no evidence of previous malignancy but presented with acute flank pain and was found to have complete ureteral obstruction.

## 2. Case Presentation

Our patient is a 42‐year‐old female at the time of presentation. She presented for cramping abdominal pain at an outside hospital, and there was a concern for a recently passed ureteral stone, given hydronephrosis without an identifiable calculus. Due to her initial presentation at an outside hospital, the current report was unable to retrieve initial laboratory findings. After this acute presentation, the patient continued to have pain and underwent a cystoscopy with left retrograde pyelogram that demonstrated a left ureteral stricture. This was balloon dilated, and a ureteral stent was placed.

The patient tolerated the ureteral stent very poorly, and the decision was made to remove the stent and place a percutaneous nephrostomy tube. Prior to nephrostomy tube placement, her creatinine was high at 1.7 mg/dL, white blood cell count 5.2 × 10^9^/L, hemoglobin 14.6 g/dL, platelets 301 × 10^9^/L, and hematocrit was low at 29.3%. Inflammatory markers and lactate dehydrogenase were not measured due to low suspicion for inflammatory conditions or cellular death. Functional testing with nuclear medicine mercaptoacetyltriglycine (MAG3) renography at this time demonstrated obstruction (the nephrostomy tube was occluded intentionally during the study) with a half‐time (T1/2) > 30 min.

**Figure 1:**
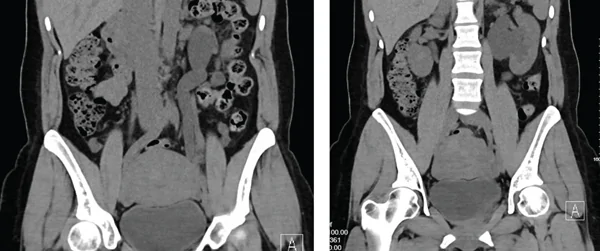
Initial CT imaging.

**Figure 2:**
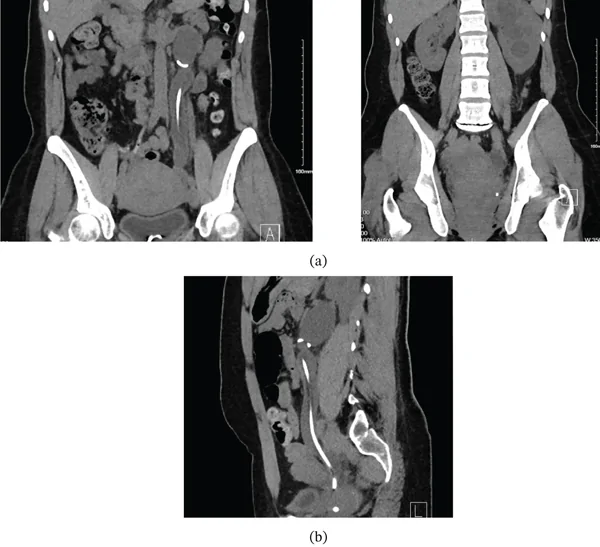
CT imaging after ureteral stent placement. (a) Coronal images after stent placement. (b) Sagittal images after stent placement.

**Figure 3:**
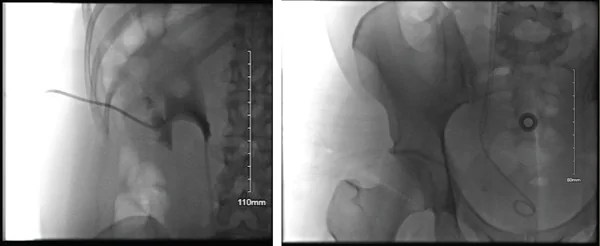
Antegrade nephrostogram at time of PCN placement.

Following a period of nephrostomy tube drainage, an antegrade nephrostogram demonstrated an obliterated ureteral stricture at the level of the pelvic brim (see Figures 1, 2, and 3). Her laboratory results for gastrointestinal and gynecologic malignancies were unremarkable. Similarly, a repeat computed tomography (CT) scan demonstrated no appreciable cause for extraluminal ureteral obstruction. The working diagnosis was either stricture from a passed stone or possible endometriosis with ureteral involvement. The differential also included urothelial carcinoma, IgG4‐related disease, and idiopathic retroperitoneal fibrosis [8, 9].

She ultimately underwent an uncomplicated ureteral reimplant with excision of scarred material outside of the distal ureter. Pathology unexpectedly demonstrated low‐grade B‐cell extranodal marginal zone lymphoma, which was the only evidence of malignancy for this patient. Immunohistochemical findings suggested that the B‐cells were positive for CD20, but were negative for CD5, CD10, CD21, CD23, CD30, CD34, BCL‐1, and BCL‐6. The CD4+ and CD8+ T cells were positive for CD3, CD5, and CD7. Ki‐67 was about 20%. The ratio for immunoglobulins IgG4/IgG was low, with MUM1 staining in some plasma cells. A clonal immunoglobulin (IGK) light chain gene rearrangement was positive.

After the diagnosis, the patient established care for her oncologic staging and management at a different institution. Due to the inability to obtain outside records for her positron emission tomography/computed tomography (PET/CT), bone marrow biopsy, oncologic treatment, and follow‐up, it was not possible to definitively determine if the current case represented primary versus secondary disease. Thus, her status clinically at the present time is not known due to her transfer to an outside hospital.

## 3. Timeline of Events

- July/August 2023: Patient presented with cramping abdominal pain. Imaging on CT revealed hydroureteronephrosis without evidence of ureteric stones.
- September 2023: Cystoscopy with retrograde pyelogram revealed left ureteral stricture in the distal/middle segments. Dilation occurred with a balloon, and a stent was placed. Later that month, the stent was removed due to severe discomfort.
- October 2023: Nephrostomy tube placed while the stent was removed. MAG3 with nephrostomy 44% with T1/2 > 30 min.
- December 2023: Antegrade nephrostogram demonstrated an obliterated ureteral stricture at the level of the pelvic brim. Gastrointestinal/gynecologic malignancy work‐up was unremarkable, with imaging showing no cause of extraluminal ureteric obstruction.
- January 2024: Uncomplicated left‐sided ureteral reimplant with pathological findings of ureteral lymphoma (low‐grade B‐cell lymphoma/extranodal marginal zone lymphoma)
- February 2024: Patient treated at an outside hospital for further oncologic management

## 4. Discussion: Epidemiology and Diagnostic Considerations

Genitourinary tract lymphomas account for a minority of extranodal lymphomas; detailed syntheses of reported ureteral lymphomas emphasize their rarity and the diagnostic challenge posed by nonspecific imaging and endoscopic appearances [1, 4, 5]. Several reports highlight the spectrum of presentation from distal ureteral strictures to pelviureteric junction obstruction, with patients presenting with flank pain, hydronephrosis, or signs of renal impairment [2, 4]. Although the case discussed refers to an isolated malignancy involving the ureter, secondary lymphoma has been noted to infiltrate the renal system [6]. In striking similarity to prior reports, the patient also presented with abdominal pain, hydronephrosis, and symptoms of postrenal obstruction that were initially puzzling on ureteric imaging, mimicking urothelial carcinoma and renal colic [7, 8].

Urothelial carcinoma often presents with hematuria, while ureteral lymphoma has been described to present without constitutional symptoms and hematuria [8]. Imaging often fails to support the diagnosis of ureteral lymphoma because it mimics urothelial carcinoma, being described as ureteral wall thickening that is concentric and homogenous in enhancement [8]. Thus, with radiological imaging modalities, findings are often nonspecific to ureteral lymphoma [7].

The differential for ureteral obstruction also included IgG4‐related retroperitoneal fibrosis, which has been shown to cause ureteral encasement on imaging and postrenal obstructive uropathy [9]. However, this disease is more likely to affect elderly males, IgG4/IgG would be above 40%, and retroperitoneal lesions would be present [9]. In addition, symptoms associated with symptomatic effect on the great vessels, kidneys, and psoas muscle would likely be present, but not all of such features were seen in the current patient [9]. A closely related idiopathic cause of retroperitoneal fibrosis was also unlikely in the patient because the disease more often affects young males, and fibrosis would be more likely on biopsy [9]. Furthermore, endometriosis with ureteral involvement was determined to be unlikely due to the lack of endometriosis at other pelvic sites like the bladder (the most common location of genitourinary migration) and gynecologic symptoms like chronic pelvic pain, dysmenorrhea, dyspareunia, and cyclical pain [10].

Likewise, diagnostic challenges in the patient′s case were similar to the existing literature, often with no definitive preoperative diagnosis prior to surgical excision [7, 8]. Histopathological and immunohistochemical analysis after surgical resection is frequently necessary for a diagnosis [7]. The existing literature suggests that lymphoma can affect various regions of the genitourinary tract and pose significant diagnostic and clinical challenges. Distinguishing primary ureteral lymphoma from secondary involvement or regional nodal disease can be nuanced; historical criteria and proposed frameworks emphasize the predominance of extranodal presentation in isolated extranodal involvement, though definitive categorization may be difficult in the setting of multifocal lymphoid disease [1, 2, 4]. Although prior case reports have noted ureteral lymphoma, primarily with reports of being mainly diffuse large B‐cell or with transformation from marginal zone lymphoma, the present case expands the literature on being a marginal zone lymphoma causing a focal ureteral obstruction without renal or bladder involvement [6–8]. Ureteral lymphoma should be on the differential in cases of obstructive uropathy, especially when endoscopic evaluation, imaging modalities, or biopsy are not able to support a leading diagnosis [7, 8]. The case is limited due to its low generalizability and lack of follow‐up.

## 5. Discussion: Pathophysiology and Mechanisms of Obstruction

Lymphomatous involvement of the ureter can arise via direct mural infiltration, extrinsic compression from retroperitoneal lymphadenopathy, or less commonly, contiguous spread from adjacent retroperitoneal disease. This array of mechanisms has been described across case reports, stressing that obstruction may result from intrinsic ureteral wall infiltration or extrinsic mass effect, with clinical consequences ranging from reversible obstruction after systemic therapy to persistent renal impairment necessitating drainage or surgical intervention [1–4].

## 6. Conclusion

B‐cell lymphoma remains a very rare cause of ureteral obstruction. Our case comprises part of the very limited data on urinary tract obstruction due to atypical malignancy. When evaluating recurrent or unexplained ureteral obstruction in a young patient, atypical malignancy such as ureteral lymphoma should be considered in the differential. It is vital to consider various uncommon or rare etiologies, especially when diagnostic work‐up is unrevealing in cases of obstruction within the urinary system. Histopathological evaluation is sometimes needed for proper diagnosis. Awareness of its diagnostic challenges and differentiating features from other common conditions can expedite early recognition of the disease and appropriate management.

## Author Contributions

All authors were engaged in the review of the case, as well as the manuscript preparation and editing.

## Funding

No funding was received for this manuscript.

## Consent

Patient information was sufficiently anonymized. Written informed consent was obtained.

## Conflicts of Interest

The authors declare no conflicts of interest.

## Supporting information


**Supporting Information** Additional supporting information can be found online in the Supporting Information section. Supporting Information. File S1: The completed CARE checklist has been submitted in accordance with the journal.

## Data Availability

Data sharing not applicable to this article as no datasets were generated or analysed during the current study.
