# Supplementary material for: Lymphoma as a Rare Cause of Ureteral Obstruction: A Case Report
Source: Case Rep Urol. 2026 Aug 21;2026:1563744. doi: 10.1155/criu/1563744 (PMC13498890; doi:10.1155/criu/1563744)
Supplement: Supplementary file 1 [file CRIU-2026-1563744-s001.pdf]

CARE case report guidelines

CARE Checklist of information to include when writing a case report

(cc) BY-NC-ND

Topic	Item	Checklist item description	Reported on Line
Title	1	The diagnosis or intervention of primary focus followed by the words "case report"	Line 1
Key Words	2	2 to 5 key words that identify diagnoses or interventions in this case report, including "case report"	line 163
Abstract (no references)	3a	Introduction: What is unique about this case and what does it add to the scientific literature?	line 30
	3b	Main symptoms and/or important clinical findings	line 36
	3c	The main diagnoses, therapeutic interventions, and outcomes	line 30, 57, 65
	3d	Conclusion—What is the main "take-away" lesson(s) from this case?	line 142, 143
Introduction	4	One or two paragraphs summarizing why this case is unique (**may include references**)	line 121-127
Patient Information	5a	De-identified patient specific information.	all de-identified
	5b	Primary concerns and symptoms of the patient.	line 36, 37
	5c	Medical, family, and psycho-social history including relevant genetic information	line 31, 32
	5d	Relevant past interventions with outcomes	line 31, 32
Clinical Findings	6	Describe significant physical examination (PE) and important clinical findings.	line 35 -53
Timeline	7	Historical and current information from this episode of care organized as a timeline	line 71 - 84
Diagnostic Assessment	8a	Diagnostic testing (such as PE, laboratory testing, imaging, surveys).	line 35-53
	8b	Diagnostic challenges (such as access to testing, financial, or cultural)	line 38 - 53
	8c	Diagnosis (including other diagnoses considered)	line 58, 59
	8d	Prognosis (such as staging in oncology) where applicable	line 65 - 70
Therapeutic Intervention	9a	Types of therapeutic intervention (such as pharmacologic, surgical, preventive, self-care)	line 65 - 70
	9b	Administration of therapeutic intervention (such as dosage, strength, duration)	line 65 - 70
	9c	Changes in therapeutic intervention (with rationale)	line 65 - 70
Follow-up and Outcomes	10a	Clinician and patient-assessed outcomes (if available)	line 65 - 70
	10b	Important follow-up diagnostic and other test results	line 65 - 70
	10c	Intervention adherence and tolerability (How was this assessed?)	line 65 - 70
	10d	Adverse and unanticipated events	line 65 - 70
Discussion	11a	A scientific discussion of the strengths AND limitations associated with this case report	line 121 - 127
	11b	Discussion of the relevant medical literature **with references**.	line 86 - 127
	11c	The scientific rationale for any conclusions (including assessment of possible causes)	line 136 - 143
	11d	The primary "take-away" lessons of this case report (without references) in a one paragraph conclusion	line 136-143
Patient Perspective	12	The patient should share their perspective in one to two paragraphs on the treatment(s) they received	line 65 - 70
Informed Consent	13	Did the patient give informed consent? Please provide if requested	Yes ☑ No ☐
